# SKLB023 Blocks Joint Inflammation and Cartilage Destruction in Arthritis Models via Suppression of Nuclear Factor-Kappa B Activation in Macrophage

**DOI:** 10.1371/journal.pone.0056349

**Published:** 2013-02-19

**Authors:** Caifeng Xie, Liang Ma, Juan Liu, Xiuxia Li, Heying Pei, Mingli Xiang, Lijuan Chen

**Affiliations:** State Key Laboratory of Biotherapy, West China Medical School, Sichuan University, Chengdu, China; Faculté de médecine de Nantes, France

## Abstract

Rheumatoid arthritis (RA) is the most common arthritis and is mainly characterized by symmetric polyarticular joint disorders. Our previous study demonstrated a novel small molecule compound (Z)-N-(3-Chlorophenyl)-2-(4-((2,4-dioxothiazolidin-5-ylidene) methyl) phenoxy) acet-amide (SKLB023) showed potently anti-arthritic effects in a rat arthritis model, however, the underlying mechanisms for this are largely unknown. Both NF-*κ*B and macrophages were reported to play important roles in the pathologic processes of RA. The purposes of this study were to indicate whether NF-*κ*B and macrophages contributed to anti-arthritic effects of SKLB023 in two experimental arthritis models. Our results showed that SKLB023 could significantly improve joint inflammation and cartilage destruction both in adjuvant induced arthritis (AIA) and collagen-induced arthritis (CIA) models. We further found that the binding activation of NF-*κ*B to DNA in joint tissues and RAW264.7 macrophages were suppressed by SKLB023. SKLB023 also inhibited the NF-*κ*B activity in peritoneal macrophages by luciferase assay. Furthermore, the number of macrophages in synovial tissues was decreased after the treatment of different doses of SKLB023. The levels of TNF-α, IL-1β, and IL-6 in plasma, and the levels of TNF-α, NO, and IL-1β in peritoneal macrophages were down-regulated by SKLB023. Finally, SKLB023 attenuated the expression of iNOS and COX-2 in vivo and suppressed the phosphorylations of components of the mitogen-activated protein kinases (MAPKs). These observations identify a novel function for SKLB023 as an inhibitor of NF-*κ*B in macrophages of RA, highlighting that SKLB023 was a potential therapeutic strategy for RA.

## Introduction

Rheumatoid arthritis (RA), which afflicts about 1% of the world's population, is a systemic, chronic, autoimmune inflammatory disease that preferentially attacks the synovial lining cells of the joints, destroys local articular structures, and further affects related tissues and organ systems [1], [2]. Therefore, therapy to joint inflammation and cartilage destruction is a logical strategy for preventing the progression of RA [3], [4].

In RA, the joint synovial tissue is abnormally infiltrated by a variety of specific cells such as macrophage, T cell, B cell, granulocyte, and monocyte [5]. Among these inflammatory cells, macrophages are abundantly present in the inflamed synovial membrane and at the cartilage-pannus junction, contributing to joint inflammation and cartilage destruction in the acute and chronic progression of RA [6]–[8]. The infiltration/activation of macrophages stimulates the over-expression of several proinflammatory cytokines including TNF-α, IL-1β, and IL-6 which are, at least in part, dependent on the activation of NF-*κ*B [9]. Additionally, large numbers of synovial lining macrophages in RA represent a source of nitric oxide (NO) that promote the highly expression of TNF-α production and further induce the incidence of synovitis [10], and the transcription of proinflammatory cytokine genes in lipopolysaccharide (LPS)-stimulated macrophages is usually regulated by the activation of NF-*κ*B signaling pathway [11].

The activation of members of NF-*κ*B transcription family is proven to be a crucial factor for contributing to chronic inflammatory responses in the pathogenesis of RA [12]–[14]. NF-*κ*B is highly activated in synovial tissue of patient with RA [15], [16] and NF-*κ*B binds to DNA sequence and further induces the transcription and production of cytokines, chemokines, and inducible nitric oxide [17], [18]. Besides, activation of NF-*κ*B usually has great effect on other signal transduction proteins, many of which are relevant to RA, including iNOS, COX-2 and components of the mitogen-activated protein kinase (MAPK) [19], [20]. Thus, the modulation of NF-*κ*B pathways may be effective approaches for the treatment of RA and other chronic inflammatory disorders.

In the present study, we demonstrate that systemic administration of SKLB023 effectively ameliorates the chronicity and progression of RA both in AIA and CIA experimental models. The potential mechanisms of SKLB023 in RA may be inhibiting the activation of NF-*κ*B signaling pathways in macrophages.

## Materials and Methods

### Ethics and Statement

Animal experiments were carried out in strict accordance with the recommendations in the Guide for the Care and Use of Laboratory Animals of the National Institutes of Health. The protocol was approved by our Institutional Animal Care and Use Committee of the Sichuan University in China (IACUC number: 20100318). All surgery was performed under chloral hydrate anesthesia, and all efforts were made to minimize suffering.

### Reagents, Cell Line and Cell Culture

SKLB023 was prepared as previously described [21]. Indomethacin, LPS (*Escherichia coli*, O111: B4), and other analytical grade chemicals were purchased from Sigma. RAW264.7 cell line was obtained from the American Type Culture Collection (TIB 71, Rockville, MD, USA). Dulbecco’s modified Eagle medium (DMEM), penicillin, streptomycin, and fetal bovine serum were purchased from Gibco Life Technologies (Rockville, MD, USA). RAW264.7 cells were grown in DMEM with 10% fetal bovine serum, 100 U/ml penicillin, and 100 µg/ml streptomycin at 37°C in a humidified atmosphere of 5% CO2 in air.

### AIA Model in Lewis Female Rats

Seven-week-old female Lewis rats were purchased from Tengxin Laboratory Animal Co. Ltd. (Chongqing, China). Freund’s complete adjuvant (CFA, Chondrex) was prepared at 10 mg/ml by suspending heat-killed *Mycobacterium tuberculosis (Mt) H37Ra* (Difco, Detroit, USA) in incomplete Freund’s adjuvant (IFA, Chondrex). Lewis female rats were injected subcutaneously at the tail base with a 100 µl of CFA emulsion, and randomly divided into five groups with each containing 8 animals. Treatment was initiated on day 14 after the immunization. Different dosages of SKLB023 suspended in 1.0% methylcellulose/PBS (10, 20, 50 mg/kg) were daily administrated by oral gavage (o.g.). The clinical score for the progression of AIA was examined every three day and the scoring for each limb ranged from 0 to 4 (0 =  no arthritis; 1 =  redness or swelling of one toe/finger joint; 2 =  redness and swelling of more than one toe/finger joints; 3 =  involvement of the ankle and tarsal-metatarsal joints; 4 =  redness or swelling of the entire paw). The arthritis score was calculated by summing the scores from all paws [22]. Animals were sacrificed on day 28, and the plasma samples were collected and the concentration of TNF-α, IL-1β, and IL-6 was determined using commercial ELISA kits (R&D Systems).

### CIA Model in DBA/1J Male Mice

DBA/1J male mice (6–7 weeks old) were obtained from Shanghai SLAC Laboratory Animal Co. Ltd (Shanghai, China). CIA was induced by subcutaneous injection with a 100 µL emulsion which contained 100 µg of bovine CII (Chondrex) and 100 µg of CFA (2 mg/ml) at the base of the tail. On day 21, 50 µl of booster emulsion containing 50 µg of bovine CII and IFA was injected subcutaneously at the tail, but at a different location from the site of first injection. Mice were randomly divided into four groups with each containing 8 animals and then treated with 2.5 mg/kg Indomethacin (Indo), 10 mg/kg, and 20 mg/kg SKLB023, respectively. The treatment continued for 27 days, during which the arthritis index was measured every three days. To determine the arthritis index, each paw was graded on a scale of 0–4 (0 =  no visible signs; 1 =  edema and erythema of a single joint or digit; 2 = 2 joints; 3 =  more than 2 joints; and 4 =  severe arthritis of the entire paw). The arthritis score was calculated by summing the scores from all paws [23]. Animals were all sacrificed on day 67, and the plasma samples were collected. The serum concentration of TNF-α, IL-1β, and IL-6 was analyzed using ELISA kits (R&D System).

### Histologic Examination and Immunohistochemical Analysis

Both legs and hind paws of Lewis rats and DBA/1J mice were removed, fixed with 4% paraformaldehyde in PBS, decalcified for 15 days with EDTA, and then embedded in paraffin. The paraffin sections were stained with H&E and Safranin O-fast green. For the immunohistochemical analysis, the ankle joints were cut into 5 µm sections and fixed in cold acetone for 20 min. Endogenous peroxidase was quenched with 3% H_2_O_2_ for 5 min. Sections were pretreated with 3% goat serum for 1 h at 37°C before the application of primary antibody. Indirect immunoperoxidase staining was performed at 37°C for 1 h. Specimen was treated with anti-CD68 monoclonal antibodies (Cell Signaling Technology, Beverly, MA) to identify the synovial macrophages.

All sections were evaluated histologically by two independent observers. For H&E, the gradation of arthritis was scored from 0 to 4 according to the intensity of lining layer hyperplasia, mononuclear cell infiltration, and pannus formation, as described previously [24]: 0, normal ankle joint; 1, normal synovium with occasional mononuclear cells; 2, definite arthritis, a few layers of flat to rounded synovial lining cells and scattered mononuclear cells; 3, clear hyperplasia of the synovium with three or more layers of loosely arranged lining cells and dense infiltration with mononuclear cells; 4, severe synovitis with pannus and erosions of articular cartilage and subchondral bone. Safranin O staining was scored with a semiquantitative scoring system (0–3), where 0 represents no loss of proteoglycans and 3 indicates complete loss of staining for proteoglycans [25]. For immunohistochemical staining, expression of the CD68 in the synovial tissue of all ankle joints present was scored semiquantitatively on a 5-point scale [26]. A score of 0 represented minimal expression, while a score of 4 represented abundant expression of a marker.

### Electrophoretic Mobility Shift Assay

Nuclear proteins were extracted from AIA joint tissues and RAW264.7 cells incubated with LPS (1 µg/ml) and 20 µM of SKLB023 for 10, 30, and 60 min as previously described [27]. The following oligonucleotides were used: NF-*κ*B sense, 5-AGCTTCAGAGGGGACTTTCCGAGAGG-3, and NF-*κ*B, antisense 5-TCGACCTCTCGGAAAGTCCCCTCTGA-3. Gel mobility-shift assay and super shift assay were performed as described [28].

### NF-κB luciferase Reporter Assay

NF-*κ*B-luc transgenic mice on a Balb/c background (Xenogen Corp., Alameda, CA) were injected with 2–3 ml of 3% thioglycollate and peritoneal exudated cells were harvested after 3 days. Peritoneal macrophages were adhered onto six-well plates (2×10^6^ cells) for 3 h. Non-adherent cells were removed, and then the attached cells were cultured with LPS (1 µg/ml) alone or with 20 µM of SKLB023 for 10, 30, and 60 min [29]. Activated macrophages were harvested and lysed in lysis buffer, and then lysate was used in accordance with luciferase assay kit (E1500, Promega, Madison, WI).

### Isolation of Peritoneal Macrophages and Cytokine Analyses in vitro

BALB/c mice were injected with 2–3 ml of 3% thioglycollate and peritoneal exudated cells were harvested after 3 days. Peritoneal macrophages were adhered onto six-well plates (2×10^6^ cells) for 3 h. Non-adherent cells were removed, and then the attached cells were cultured with DMEM medium alone or activated with LPS (1 µg/ml) and 20 µM of SKLB023 for18 h. Culture supernatants were analyzed for NO using the Greiss reagent, and for TNF-α, IL-1β, and IL-6 using ELISA kits (R&D Systems, Minneapolis, MN) as recommended by the manufacturer.

### Western Blot Analysis

RAW264.7 macrophages (ATCC), were cultured in 6-well plates (1×10^6^ cells/well) overnight, and then incubated with LPS (1 µg/ml) and 20 µM of SKLB023 for 10, 30, and 60 min. Cells were washed with PBS, and lysed in lysis buffer containing 0.1% Triton X-100, 20 mM Tris (pH 8.0), 100 mM KCl, 1 mM dithiothreitol, 1 mM phenylmethylsulfonyl fluoride, and protease inhibitor cocktail. Total cell lysates were electrophoresed, and transferred to PVDF membrane. The membrane was incubated with antibodies overnight at 4°C, and 1 h at room temperature with anti-rabbit or anti-mouse secondary antibodies linked to horseradish peroxidase. Proteins were visualized using the chemiluminescence system (Bio-Rad) and autoradiography. Proteins extracted from the ankles of AIA and CIA was subjected to 8% SDS-PAGE and then incubated with anti-iNOS (1∶1000), anti-COX-2 (1∶1000), and anti-β-actin (1∶1000) antibodies (Cell Signaling Technology, Beverly, MA) for 60 min.

### Statistical Analysis

Data are presented as means ± s.d. for each group. The Mann–Whitney U test was used for comparison of medians from independent samples, in all cases, *P* values less than 0.05 were considered significant.

## Results

### SKLB023 Ameliorates AIA

**Figure 1:**
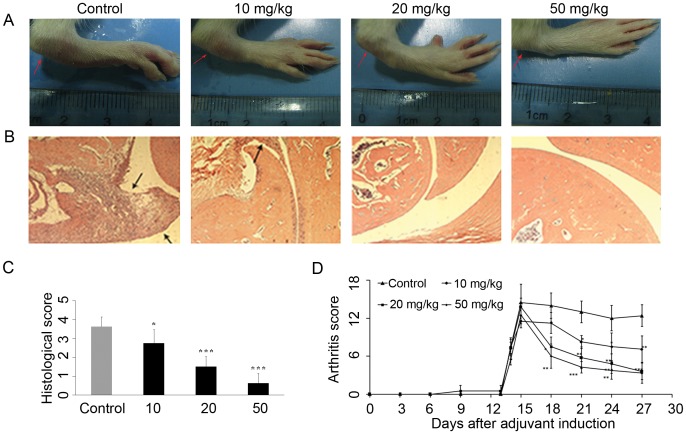
SKLB023 improved histological changes and arthritis scores in Lewis rats. (A) Representative photographs of tarsotibial joint swelling of the hind paws in rats treated with or without SKLB023 in AIA (n = 8). (B) Representative histopathologies of the ankle joints stained with hematoxylin and eosin (H&E) in AIA. (C) Histological examinations of a total of 8 rats for each pair were scored from 0 to 4 as described in Materials and Methods. (D) Arthritis scores during the progression of AIA. Error bars represented SEM, *P<0.05, **P<0.01, and ***P<0.001 indicated significant differences from the control group.

To assess different doses of SKLB023 in the development of arthritis, the AIA model in Lewis female rats was initially performed. After the oral administration of SKLB023 at a dosage of 50 mg/kg for two weeks, the arthritic signs characterized by erythematous oedema appearing in limbs and joint swelling were markedly improved (**Figure 1A**). The treatment with SKLB023 contributed to a gradual suppression of these histopathological changes, and inhibited the mononuclear cell infiltration and pannus formation in synovial tissues. There was a statistically significant difference in scores for H&E staining between the SKLB023-treated group and control group (**Figure 1B, 1C**). Importantly, these treated rats access SKLB023 displayed delayed onset, lower incidence and decreased severity of AIA compared to untreated rats (**Figure 1D**). The articular scores, especially at a dose of 20 and 50 mg/kg, were both ≤ 4 at the end point of treatment (both P<0.01).

**Figure 2:**
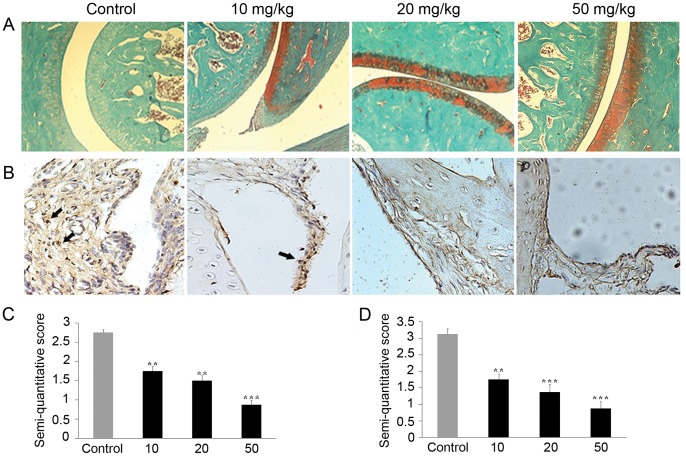
SKLB023 improved cartilage destruction and macrophages infiltration in AIA model. (A) Representative histopathologies of the ankle joints stained with Safranin-O in AIA. (B) SKLB023 inhibiting the infiltration of CD68 positive cells in synovial tissue of AIA model. Synovial tissue sections were analyzed for the expression of CD68 using immunohistochemical method as described in Materials and Methods. (C) Histologic analysis of cartilage in AIA rats after SKLB023 therapy. Sections were scored in a blinded manner on a 4-point scale as described in Materials and Methods. Significantly less loss of safranin O staining was observed in the animals treated with SKLB023 than in controls, indicating inhibition of cartilage destruction. (D) CD68 expression was measured by immunohistochemistry in rats with AIA. It was scored in a blinded manner on a scale of 0 to 4 as described in Materials and Methods. A statistically significant reduction of CD68 expression was observed in the rats treated with different doses of SKLB023. Error bars represented SEM, **P<0.01, and ***P<0.001 indicated significant differences from the control group.

The degradation and destruction of articular cartilage tissues was assessed by Safranin O-fast green staining, a method to detect the matrix proteoglycan depletion. Proteoglycan loss in the ankle joints of groups treated with SKLB023 was significantly decreased than that of control groups (**Figure 2A, 2C**), stating that the cartilage profiles in RA have been significantly improved after the daily administration of SKLB023 as illustrated by the increased deep red Safranin O staining in cartilage tissues (**Figure 2A**).

CD68 is the major phenotype of macrophage-like synovial cells which played a critical role in the progression of RA. Representative analysis of the infiltration of CD68 positive cells in synovial tissue of AIA rats were displayed using immunohistochemical methods (**Figure 2B**). Large numbers of CD68 positive cells in the control groups were detected in the lining layers of synovium. In contrast, the number of CD68 positive cells in SKLB023-treated groups was markedly decreased to 52.0% (P<0.01), 43.4% (P<0.001), and 24.9% (P<0.001) at a dose of 10, 20, and 50 mg/kg, respectively (**Figure 2D**).

### SKLB023 Ameliorates CIA

**Figure 3:**
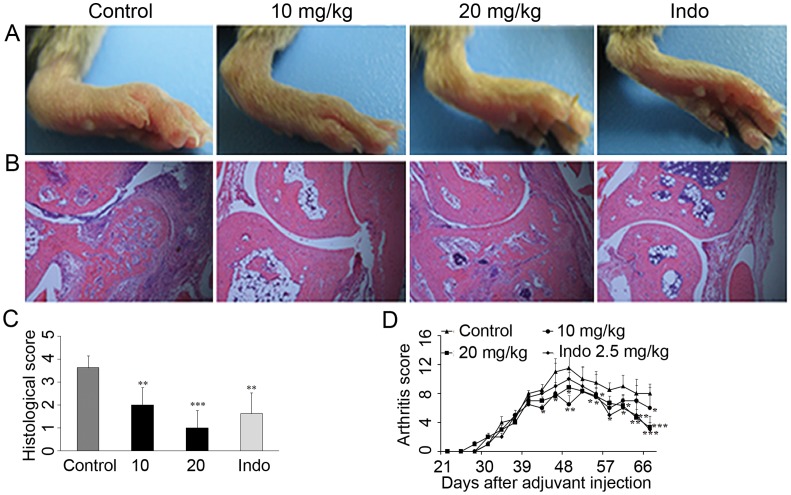
SKLB023 improved histological changes and arthritis scores in DBA/1J mice. (A) Representative photographs of tarsotibial joint swelling of the hind paws in mice treated with or without SKLB023 (n = 8). (B) Representative histopathologies of the ankle joints stained with hematoxylin and eosin (H&E) in CIA. (C) Histological examinations of a total of 8 mice for each pair were scored from 0 to 4 as described in the Methods. (D) Arthritis scores during the progression of CIA. Error bars represented SEM, *P<0.05, **P<0.01, and ***P<0.001 indicated significant differences from the control group.

**Figure 4:**
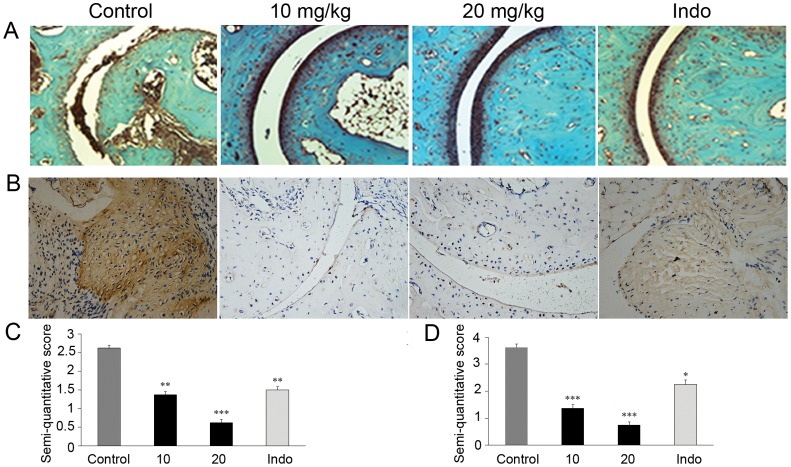
SKLB023 improved cartilage destruction and macrophages infiltration in CIA model. (A) Representative histopathologies of the ankle joints stained with Safranin-O in CIA. (B) SKLB023 inhibiting the expression of CD68 in synovial tissue of CIA model. Synovial tissue sections were analyzed for the expression of CD68 using immunohistochemical method as described in Materials and Methods. (C) Histologic analysis of cartilage in CIA mice after SKLB023 therapy. Sections were scored in a blinded manner on a 4-point scale as described in Materials and methods. Significantly less loss of safranin O staining was observed in the animals treated with SKLB023 than in controls, indicating inhibition of cartilage destruction. (D) CD68 expression was measured by immunohistochemistry in rats with CIA. Expression of CD68 was scored in a blinded manner on a scale of 0 to 4 as described in Materials and Methods. A statistically significant reduction of CD68 expression was observed in the rats treated with different doses of SKLB023.Error bars represented SEM, *P<0.05, **P<0.01, and ***P<0.001 indicated significant differences from the control group.

Previously, 20 mg/kg of SKLB023 exhibited similar activity to 50 mg/kg of that in AIA model. And then, the CIA model in DBA/1J male mice was further evaluated for the effects of SKLB023 on preventing autoimmune arthritis at the dosages of 10, and 20 mg/kg. Representative photographs of the tarsotibial joint swelling of the hind paws from DBA/1J mice were shown in **figure 3A**. 20 mg/kg/day of SKLB023 inhibited the erythematous oedema in CIA mice, which was comparable to indomethacin at a dose of 2.5 mg/kg/day. H&E staining of the ankle joints indicated that administration of SKLB023 at a dose of 20 mg/kg resulted in a significant suppression of these histopathological changes compared to control group (**Figure 3B, 3C**). These SKLB023-treated mice (20 mg/kg) exerted similar therapeutic results to the ones treated with indomethacin (2.5 mg/kg). However, a 10 mg/kg/day dose of SKLB023 reduced the arthritic severity, but did not block the arthritic onset (**Figure 3D**). Additionally, the SKLB023-treated CIA mice at a dose of 20 mg/kg approximately recovered to the normal levels by the analysis of Safranin O-fast green staining (**Figure 4A, 4C**).

Representative analysis of the infiltration of CD68 positive cells in synovial tissue of CIA mice were presented in **figure 4A**. Mice treated with 10, and 20 mg/kg SKLB023 decreased the infiltration of CD68 positive cells to respective 51, and 19% compared to untreated mice, while mice treated with indomethacin only decreased to 62%.

### SKLB023 Inhibited Proinflammatory Cytokines Production in vivo

**Figure 5:**
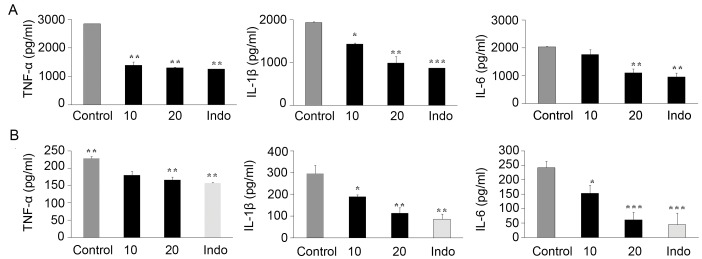
SKLB023 decreased the level of proinflammatory cytokines in two animal models of arthritis. (A) Serum levels of TNF-α, IL-6, and IL-1β in AIA. (B) Serum levels of TNF-α, IL-6 and IL-1β in CIA. Error bars represented SEM, *P<0.05, **P<0.01, and ***P<0.001 indicated significant differences from the control group.

As shown in **figure 5A**, the serum levels of TNF-α, IL-1β, and IL-6 in AIA rats were significantly increased compared to the control ones while the levels (especially IL-1β) were decreased in a dose-dependent manner after the treatment with SKLB023 (10, 20, and 50 mg/kg). In addition, the plasma concentrations of these proinflammatory cytokines were also effectively down-regulated by SKLB023 and close to the ones of indomethacin in CIA model (**Figure 5B**).

### SKLB023 Suppressed the Activation of NF-κB Both in vivo and in vitro

**Figure 6:**
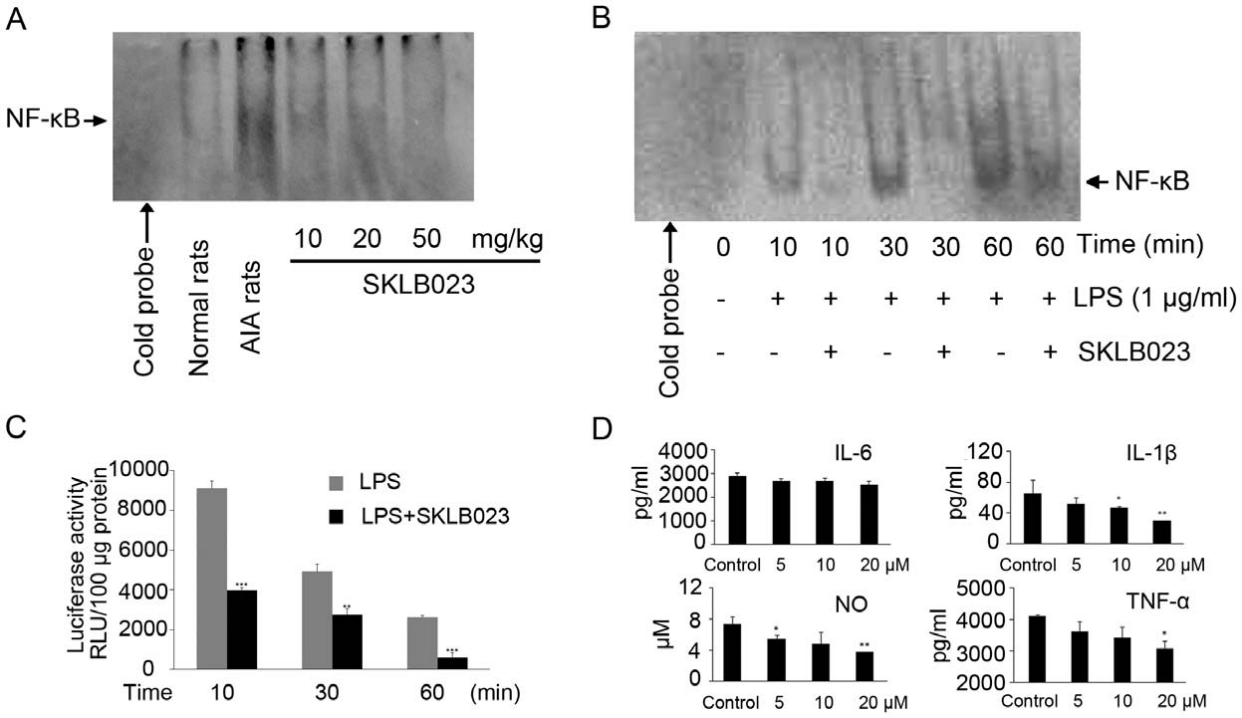
SKLB023 suppressed the activity of NF-κB both in vivo and in vitro. (A) EMSA assay using an NF-κB probe on nuclear extracts of each group rats, different doses of SKLB023 blocked the binding of DNA and NF-κB in vivo. (B) EMSA assay using an NF-κB probe on nuclear extracts of RAW264.7 cells stimulated with LPS (1 µg/ml) and incubated with SKLB023 (20 µM) for the indicated time. (C) Luciferase activity in extracts of peritoneal macrophages from NF-κB luciferase transgenic mice incubated with LPS(1 µg/ml)and then treated with SKLB023(20 µM) for 10,30 and 60 min. (D) Peritoneal macrophages from BALB/c mice were not activated or activated with LPS/IFN-γ, and then cocultured with SKLB023. Culture supernatants were assayed for TNF-α, IL-6, and IL-1β by ELISA kits, and NO production by Greiss assay. Error bars represented SEM, *P<0.05, **P<0.01, and ***P<0.001 indicated significant differences from the control group.

To confirm that SKLB023 blocked NF-κB activity in vivo, we carried out electrophoretic mobility shift assay (EMSA) using tissue samples prepared from the ankle joints of the AIA mice. NF-κB DNA-binding activity was decreased in the joints of rats treated with different dosages of SKLB023, in contrast to untreated rats (**Figure 6A**).

To determine the effects of NF-κB inhibition by SKLB023 on RA in vitro, we obtained the nuclear extracts from LPS-stimulated RAW264.7 cells in the presence or absence of SKLB023. The EMSA results showed that LPS could significantly increase the NF-*κ*B-DNA binding affinity at 10, 30, and 60 min, whereas the binding activity was suppressed by the pretreatment of SKLB023 at the concentration of 20 µM (**Figure 6B**). The densitometry data for both in vivo and in vitro NF-κB-DNA binding affinity were showed in **Figure S1**. Furthermore, SKLB023 dose-dependently inhibited LPS-induced NF-*κ*B transcriptional activity in peritoneal macrophage derived from NF-*κ*B-luciferase transgenic mice (**Figure 6C**).

### SKLB023 Decreased Proinflammatory Cytokines Production in vitro

Our previous studies indicated that SKLB023 inhibited the production of NO in RAW264.7 cells. In present investigation, we evaluated the in vitro anti-inflammatory effects of SKLB023 on the activation of peritoneal macrophage by ELISA analysis (**Figure 6D**). We demonstrated that the inducible production of several important proinflammatory cytokines such as TNF-α, IL-1β, and NO were reduced by SKLB023 in a dose-dependent manner, whereas IL-6 seemed to be not sensitive to SKLB023 in vitro.

### SKLB023 Attenuated the Expression of iNOS and COX-2 and the Phosphorylations of MAPKs

**Figure 7:**
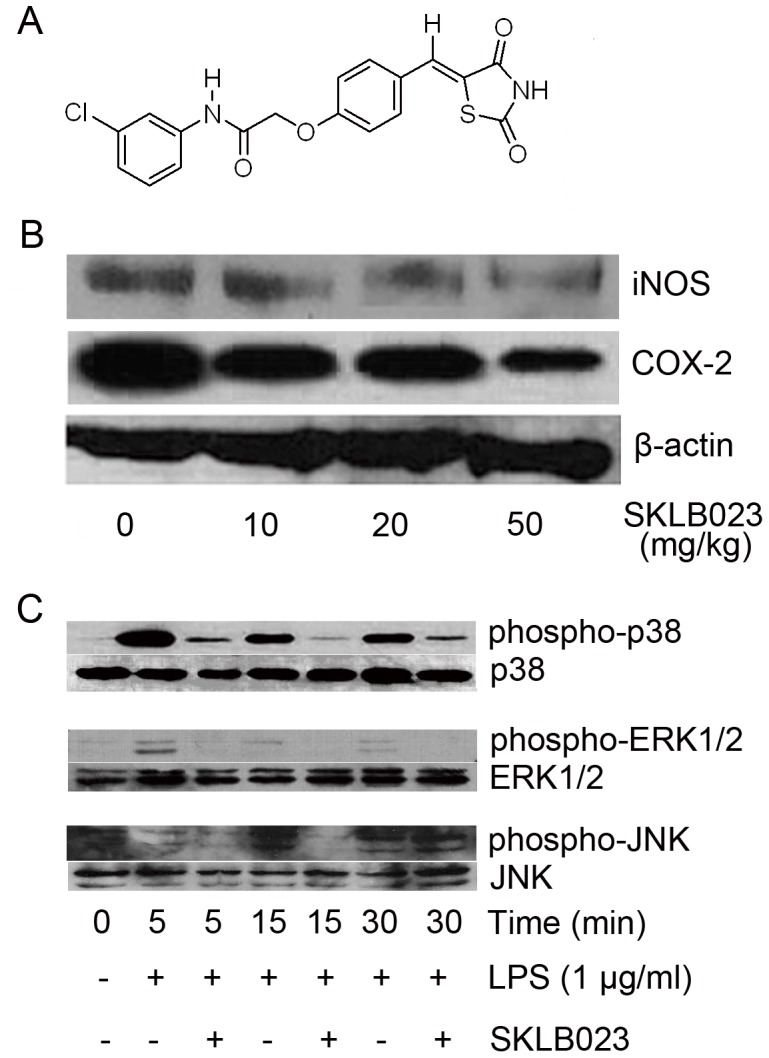
SKLB023 inhibited the phosphorylation of MAPKs. (A) Chemical structure of SKLB023. (B) iNOS and COX-2 expression in the ankle joints of AIA rats untreated or treated with various doses of SKLB023. (C) RAW264.7 cells were stimulated with LPS (1 µg/ml) and incubated with SKLB023 (20 µM) for the indicated time. Cell lysates were prepared and blotted with total or phosphospecific antibodies to ERK1/2 (Thr202/Tyr204), p38 MAPK (Thr180/Tyr182), and JNK (Thr183/Tyr185).

As the activation of NF-κB plays a crucial role in LPS-induced transcriptional regulation of iNOS and COX-2 gene expressions in macrophages, we determined whether SKLB023 inhibited the expressions of iNOS and COX-2 proteins in AIA rats. In normal Lewis rats, iNOS and COX-2 proteins were rare and even undetectable. Importantly, SKLB023 (**Figure 7A**) dose-dependently blocked the iNOS and COX-2 protein expressions (**Figure 7B**), stating that the down-regulation of iNOS and COX-2 proteins by SKLB023 were favorable for the treatment of RA in rats.

Activation of MAPKs pathway is widely involved in aberrant inflammatory responses. Thus, the total expression of MAPKs proteins (ERK1/2, p38 MAPK, and JNK) and their corresponding phosphoproteins (p-ERK1/2, p-p38 MAPK, and p-JNK) were subsequently analyzed by Western blot. Previous report demonstrated that the maximal levels of MAPKs phosphoproteins expressed during 30 min after the stimulation of LPS in human and murine monocytes/macrophages [30]. In our study, RAW264.7 cells were pretreated with SKLB023 at a concentration of 20 µM for 30 min, stimulated by LPS (1 µg/ml, for 0, 5, 10, and 30 min), and then performed for the analysis of MAPKs activation. As depicted in **figure 7C**, the phosphorylated effects of ERK1/2, p38 MAPK, and JNK were gradually up-regulated by the induction of LPS. However, SKLB023 attenuated the phosphorylations of ERK1/2, p38 MAPK at 5, 10, and 30 min, and of JNK at 10 min, suggesting that SKLB023 attenuated the expression of the phosphorylations of MAPKs.

## Discussion

In this study, we have shown that SKLB023 improved the severity of RA in a dose-dependent manner in two animal models of arthritis. We found that SKLB023 decreased the number of macrophages in the synovial tissues and suppressed the activity of NF-*κ*B in AIA rats. We also demonstrated that SKLB023 blocks joint inflammation and cartilage destruction in arthritis models via suppression of nuclear factor-kappa B activation in macrophage.

Activation of macrophages plays a pivotal role in the development and progression of joint inflammation and cartilage destruction in RA. And further these activated cells secreted high levels of proinflammatory cytokines (NO, IL-1β, TNF-α, and IL-6) in the joint synovial tissues of RA [31]. Our studies have shown that SKLB023 suppressed the infiltration of CD68 (macrophage marker) positive cells in ankle joints of AIA rats and CIA mice. Besides, all the serum levels of IL-1β, TNF-α and IL-6 were decreased by the treatment with different doses of SKLB023 compared to those of the control groups in vivo. Thus, SKLB023 could protect joints from inflammation through its influence on macrophages. However, SKLB023 significantly inhibited the expression of IL-1β, TNF-α, NO but not IL-6 in peritoneal macrophages in vitro. This may indicate that SKLB023 inhibited the IL-6 production in a different pathway responsible for the in vivo effects.

NF-*κ*B could regulate cell proliferation, apoptosis, cytokine expression, and metalloproteinase production. In addition, they modulate various genes through both transcriptional and post-transcriptional mechanisms in RA and play key roles in adaptive immune inflammatory responses [32], [33]. The functions of NF-*κ*B suggest that the inhibition of NF-*κ*B activity would reduce the production of proinflammatory cytokines and further modulate the related inflammatory reactions [34], [35]. Therefore, an alternative targeting to the signal pathways of NF-*κ*B was proposed [36]. In the present study, we found that SKLB023 suppressed the DNA-binding activity to NF-*κ*B in ankle joints of AIA rats. In addition, we demonstrated that the activity of NF-*κ*B also be inhibited by SKLB023 in peritoneal macrophages and macrophage cell line, RAW264.7. Furthermore, Additional activation of p38 and extracellular-signal–regulated kinase mitogen-activated protein kinases (MAPKs) was necessary to trigger destructive inflammation on top of the already active NF-κB [37]. It is interesting that we observed SKLB023 attenuated the phosphorylations of ERK1/2, p38 MAPK, and JNK in RAW264.7. NF-κB is activated in the synovium of patients with RA and regulates genes that contribute to inflammation, including iNOS and COX-2. In our previous study, SKLB023 inhibited the expression of iNOS and COX-2 in vitro. Accordingly, in our present study, we further demonstrated that SKLB023 decreased the expression of iNOS and COX-2 in vivo. These results indicated that SKLB023 not only affected the binding activity of NF-*κ*B to DNA, also the expression of proteins related with NF-*κ*B signaling pathways in RA, which warrant the amelioration of SKLB023 on the RA and other inflammatory diseases.

During the past 15 years, the improved understanding of the pathophysiology of RA has led to a series of dramatic changes in the therapeutic strategies [38]. However, severe side effects have been observed in the long-term administration of many drugs for the treatment of RA [39]. Recently, a better understanding of the signal transduction systems and gene regulation by transcription factors involved in cytokine production has opened the way for the discovery of novel therapeutic compounds useful in treating patients with RA [40], [41]. Our experiments shown that inhibition of transcription factor NF-*κ*B in the specific cell type macrophage improved the condition of RA decreased the production of cytokines and the expression of RA related proteins.

In conclusion, inhibition of NF-*κ*B in macrophages by SKLB023 ameliorated the joint inflammation and cartilage destruction in the progression of RA, and it serves as a novel therapeutic strategy for rheumatoid arthritis. Future works are required to identify how SKLB023 inhibits the NF-*κ*B signaling cascade, and how SKLB023 decrease the production of IL-6 in vivo.

## Supporting Information

Figure S1 **The densitometry data for NF-κB-DNA binding affinity.** (A) The densitometry data for NF-κB-DNA binding affinity in AIA rats. Error bars represented SEM, **P<0.01, and ***P<0.001 indicated significant differences from the AIA rats group. (B) The densitometry data for NF-κB-DNA binding affinity in RAW264.7 cells. Error bars represented SEM, *P<0.05, and ***P<0.001 indicated significant differences from the group treated with LPS alone.
(TIF)
