# Supplementary figures and images for: SKLB023 Blocks Joint Inflammation and Cartilage Destruction in Arthritis Models via Suppression of Nuclear Factor-Kappa B Activation in Macrophage
Source: PLoS One. 2013 Feb 19;8(2):e56349. doi: 10.1371/journal.pone.0056349 (PMC3576337; doi:10.1371/journal.pone.0056349)

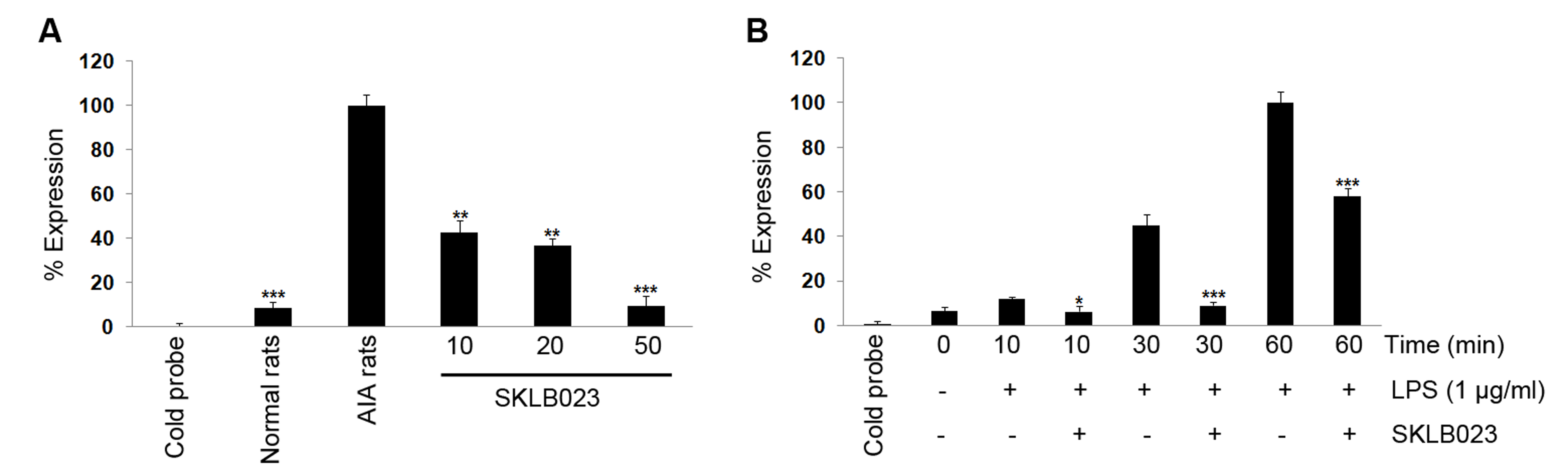

Supplement: Figure S1 — The densitometry data for NF-κB-DNA binding affinity. (A) The densitometry data for NF-κB-DNA binding affinity in AIA rats. Error bars represented SEM, **P<0.01, and ***P<0.001 indicated significant differences from the AIA rats group. (B) The densitometry data for NF-κB-DNA binding affinity in RAW264.7 cells. Error bars represented SEM, *P<0.05, and ***P<0.001 indicated significant differences from the group treated with LPS alone. (TIF) [file pone.0056349.s001.tif]
